# Future Perspectives: Therapeutic Targeting of Notch Signalling May Become a Strategy in Patients Receiving Stem Cell Transplantation for Hematologic Malignancies

**DOI:** 10.1155/2011/570796

**Published:** 2010-10-04

**Authors:** Elisabeth Ersvaer, Kimberley J. Hatfield, Håkon Reikvam, Øystein Bruserud

**Affiliations:** ^1^Division of Hematology, Department of Medicine, Haukeland University Hospital and Institute of Medicine, University of Bergen, N-5021 Bergen, Norway; ^2^Medical Department, Haukeland University Hospital, N-5021 Bergen, Norway

## Abstract

The human Notch system consists of 5 ligands and 4 membrane receptors with promiscuous ligand binding, and Notch-initiated signalling interacts with a wide range of other intracellular pathways. The receptor signalling seems important for regulation of normal and malignant hematopoiesis, development of the cellular immune system, and regulation of immune responses. Several Notch-targeting agents are now being developed, including natural receptor ligands, agonistic and antagonistic antibodies, and inhibitors of intracellular Notch-initiated signalling. Some of these agents are in clinical trials, and several therapeutic strategies seem possible in stem cell recipients: (i) agonists may be used for stem cell expansion and possibly to enhance posttransplant lymphoid reconstitution; (ii) receptor-specific agonists or antagonists can be used for immunomodulation; (iii) Notch targeting may have direct anticancer effects. Although the effects of therapeutic targeting are difficult to predict due to promiscuous ligand binding, targeting of this system may represent an opportunity to achieve combined effects with earlier posttransplant reconstitution, immunomodulation, or direct anticancer effects.

## 1. Introduction

 The most important members of the human Notch system are the four Notch receptors and their five ligands. Notch-mediated signalling is important in embryonic hematopoiesis and development of the immune system, regulation of the peripheral immune system, and development of hematological malignancies, especially T cell acute lymphoblastic leukemia (T-ALL) [1–3]. Thus, for patients treated with allogeneic stem cell transplantation for hematological malignancies, agonistic or antagonistic targeting of Notch signalling may become useful to (i) achieve more effective and safe antileukemic treatment and thereby reduce the risk of posttransplant relapse through direct targeting of the malignant cells, (ii) enhance T cell reconstitution and thereby reduce posttransplant immune defects, and (iii) develop new immunomodulatory strategies that can reduce the risk of severe infections and severe graft versus host disease (GVHD) without inhibition of graft versus leukemia (GVL) effects. Even a combination of these effects may become a possible treatment by careful selection of molecular targets.

## 2. Notch Molecules, Notch Ligands, and Downstream Signalling

### 2.1. Notch and Notch Ligands

**Figure 1:**
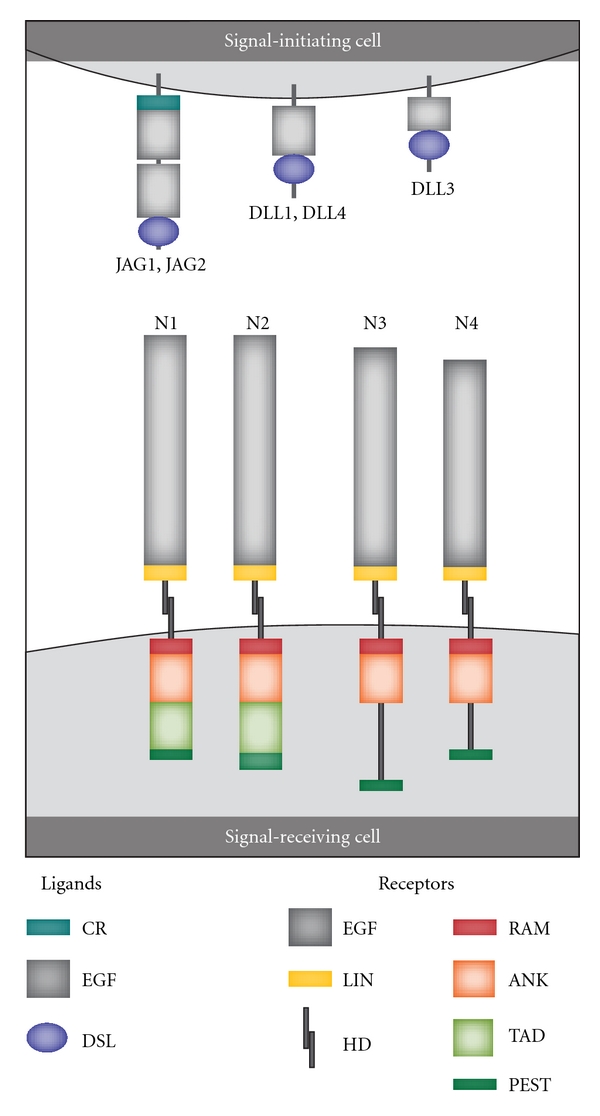
Notch receptors and their ligands. Signal-initiating cells express Notch ligands of the Delta-like (DLL1, DDL3, DLL4) or Jagged families (JAG1, JAG2). Common structural features of all ligands are the Epithelial growth factor-like (EGF) repeats and the distal amino-terminal domain called DSL (Delta, Serrate, and Lag-2). DSL is involved in receptor binding. Additionally, JAG1 and JAG2 contain a proximal cysteine-rich (CR) domain between the EGF-like repeats and the plasma membrane. In humans there are four heterodimeric Notch receptors (Notch1-4; N1-N4). The extracellular Notch receptor domain contains EGF-like repeats, a cysteine-rich LIN-12 repeats (LIN domain) that prevents ligand-independent activation, and the proximal heterodimerization domain (HD). The cytoplasmic domain contains an RBP-J-associated molecule (RAM) domain (closest to the cell membrane) followed by ankyrin repeats (ANK) that bind to the CSL (CBF1/RBP-J*κ*/Suppressor of Hairless/LAG-1) transcription factor, a transactivation domain (TAD; only Notch 1 and 2), and a PEST (proline, glutamic acid, serine, threonine) sequence that is important for stabilization of the protein (adapted from [1]).

 Humans possess the four heterodimeric transmembrane Notch receptors Notch1-4 that can bind the five transmembrane ligands Delta-like 1, 3, and 4 (DLL1/3/4) and Jagged 1 and 2 (JAG1/2) (Figure 1). The receptor chains are cleaved by a furin-like protease in the Golgi apparatus during their way to the cell surface where they form heterodimeric receptors. These receptors consist of an extracellular subunit (NEC) with a distant part with a variable number of glycosylated Epithelial growth factor (EGF-) like repeats followed by LIN domains that prevent ligand-independent activation. The transmembrane and cytoplasmic (NTM) subunit consists of the cytoplasmic RAM domain followed by ankyrine repeats that bind to the effector transcription factor CBF1, two nuclear localization signals, a transactivation domain that is present only in Notch1 and Notch2, and finally a PEST sequence involved in stabilization of the protein. 

 The five ligands also differ in their structure (Figure 1): the amino-terminal DSL domain (Delta, Serrate, and Lag-2) which is involved in receptor binding is common to all ligands; this is followed by a variable number of EGF repeats; JAG1/2 contains an additional C-terminal cysteine-rich domain (CR). The Delta ligands seem to have two activities: to *trans*-activate Notch in neighboring cells and to *cis*-inhibit Notch in its own cells [4]. Glycosylation of the extracellular Notch domain modulates ligand-initiated Notch signalling; for example, with regard to one experimental model DLL ligands were preferred over JAG ligands when the receptors contained N-Acetylglucosamine on the O-fucose residues in the EGF-like repeats [1, 5]. Notch receptors seem to be promiscuous with regard to ligand binding, although it should be emphasized that the ligand specificity of the various receptors has not been characterized in detail. Notch1, Notch2, and Notch3 can all be activated by different ligands like DLL1, JAG1/2 [6, 7]; however, as mentioned, the ligand specificity of Notch1-4 has not been characterized in detail.

### 2.2. Canonical Intracellular Signalling

**Figure 2:**
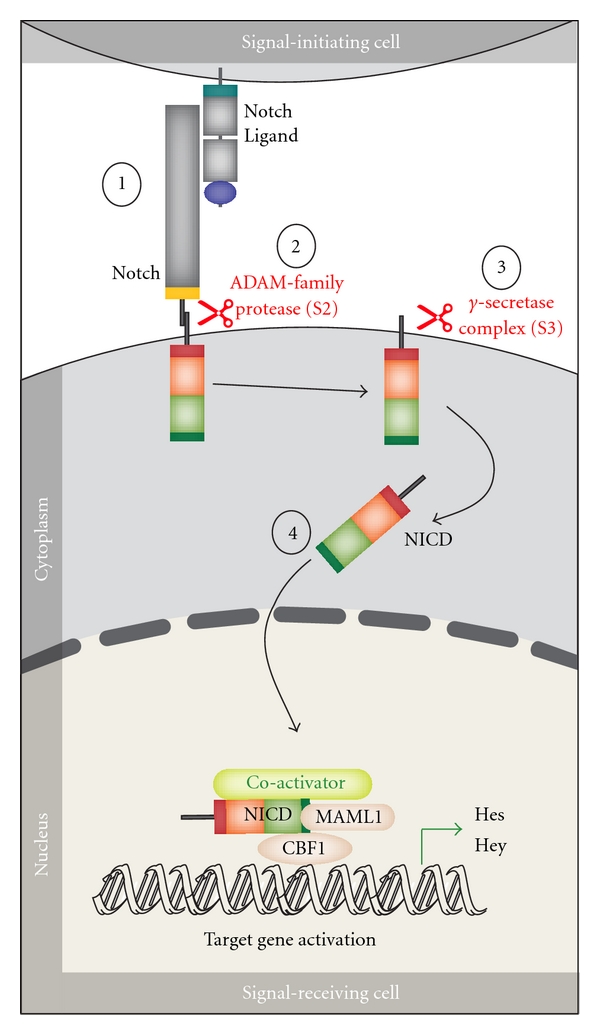
Notch canonical signalling pathway. The figure shows an overview of the canonical Notch signalling pathway (adapted from [8]). The signalling is initiated by ligand binding to Notch receptor (1). This binding initiates two subsequent proteolytic cleavages of the Notch receptor by the ADAM family protease (2) and the *γ*-secretase, respectively (3). The Notch IntraCellular Domain (NICD) is thereby released to the cytoplasm (4) and translocates to the nucleus where NICD heterodimerizes with the DNA-binding transcription factor CBF1 (also named CSL or Rbp-j). Additional coactivators are also recruited, including mastermind-like proteins (MAML1-3) ultimately leading to induction of transcriptional expression of downstream target genes, including those belonging to the Hes and Hey families.

 The first event in canonical Notch signalling (Figure 2) is ligand-receptor interaction with initiation of two successive proteolytic cleavages of the receptors (at sites S2 and S3 by ADAM family protease and the *γ*-secretase, resp.) and thereby the release of the Notch IntraCellular Domain (NICD). NICD translocates to the nucleus where it heterodimerizes with the DNA-binding transcription factor CBF1 (also named CSL or Rbp-j) and recruits other coactivators, including mastermind-like proteins (MAML1-3) and the MED8-mediator transcription activation complex; this leads to induction of transcriptional expression of target genes. The Notch-associated gene expression profile will not depend on the receptor mediating the signal only since binding of different ligands to the same receptor will in some cases have different functional effects [1, 9].

### 2.3. Noncanonical Signalling

 Noncanonical Notch signalling is well documented [2, 10], but less characterized than the canonical pathway. There are probably three types of noncanonical Notch signalling: Type I involves Notch ligation and translocation of activation signals independent of CBF1 (NICD-dependent but CBF1-independent); Type II involves activation of Notch target genes independent of S3 cleavage (NICD- and CBF1-independent); Type III involves CBF1-dependent gene activation without receptor cleavage and NICD release [10]. Several signalling pathways are involved, including Hedgehog, Jak/STAT, RTK, TGF, Wnt, PI3/Akt, mTor/Akt, JNK, MEK/ERK, and NF*κ*B [2, 10]. Noncanonical Notch signalling seems important for maintenance of lineage-restricted hematopoietic progenitors, and several of the mediators involved in this signalling are in addition important in leukemogenesis as well as regulation of cellular immune responses. The noncanonical pathway thus represents a point of crosstalk between other intracellular signalling pathways.

## 3. Notch and Hematopoietic Progenitors

### 3.1. Notch in the Hematopoietic System

 Notch1-mediated signals are essential for generation of definitive Hematopoietic stem cells (HSCs) during embryogenesis [11] though as described in more detail below, canonical Notch signalling seems to be dispensable for HSC maintenance in adults. HSCs reside primarily in the bone marrow in a complex microenvironment consisting of stromal cells, microvessels and extracellular matrix. These cells have the dual ability to self-renew as well as being able to give rise to all the cells in the hematopoietic system. Mainly HSCs are in a quiescent state; that is, the cells are in G0/G1 of the cell cycle and do not proliferate. An important factor in regulating the fate of HSCs in terms of HSC quiescence, self-renewal and differentiation, is the surrounding stem cell microenvironment, the so-called stem cell niche. HSCs have been shown to be in close proximity to cells lining the endosteum as well as near the specialised blood vessels in bone marrow called sinusoids [12].

### 3.2. The Osteoblastic Stem Cell Niche

 The *osteoblastic* niche (also referred to as the endosteal niche) [13, 14] and the *vascular* niche [12, 15] create a supportive environment for stem cells. Notch signalling is thought to be a key signalling pathway involved in maintenance and expansion of the HSC pool. In addition, an important role of Notch signalling in osteoblast and osteoclast homeostasis was recently described [16, 17]. Hematopoietic progenitor cells express Notch receptors and are exposed to Notch ligands in the bone marrow such as expression of JAG1 and DLL1 by osteoblasts [13, 18]. In a study by Calvi et al., parathyroid hormone stimulation of osteoblasts in mice resulted in induced osteoblastic proliferation with increased expression of JAG1 and a Notch1-mediated expansion of HSCs [13, 19]. These observations identified Notch as an important component of the stem cell niche that supports osteoblastic HSC regulation. However, further studies of osteoblastic regulation of HSCs via the Notch pathway have yielded conflicting results. Using serial transplantation studies, long-term reconstitution of HSCs was shown to be impaired after inhibition of Notch signalling [20]. In contrast, inactivation of neither JAG1 nor Notch1 impaired HSC maintenance in conditional knockout mouse models [21]. In a study by Maillard et al., Notch signalling was blocked by elimination of CBF1 and expression of dominant negative MAML mutants, and canonical Notch signalling was shown to be dispensable for the maintenance of long-term (LT) HSCs *in vivo* [22].

### 3.3. The Endothelial Stem Cell Niche

 Endothelial cells promote HSC expansion and self-renewal *in vitro* and are shown to have an important role in engraftment of HSCs and reconstitution of hematopoiesis *in vivo* [23]. Inhibition of VEGFR-2 signalling in sinusoidal endothelial cells impaired vascular recovery and hematopoietic reconstitution following irradiation in mice [23]. Thus, hematopoietic regeneration after myeloablation depends on vascular recovery and endothelial cell function, and Notch has been implicated in cell-cell interactions between HSCs and endothelial cells that regulate HSC function. *In vivo*, sinusoidal endothelial cells express Notch ligands JAG1 and JAG2 [24], and Notch-activated HSCs have been visualised in close proximity to the bone marrow vasculature [25]. A recent study used angiogenic models to demonstrate that Notch signalling via endothelial cells plays a role in regulation of HSCs in the vascular niche [24]. Increased expression of Notch ligand on endothelial cells after stimulation with soluble kit ligand stimulated the expansion of repopulating CD34^−^Flt3^−^cKit^+^Lineage^−^Sca1^+^ LT-HSCs at the expense of reducing differentiation, and serial transplantation assays demonstrated that these LT-HSCs retained their self-renewal ability. Furthermore, in a coculture model, endothelial cells failed to expand HSCs derived from Notch1-/Notch2-deficient mice.

### 3.4. Notch as a Part of an Interactive Cell Signalling Network

 Notch-initiated signalling is part of an interacting network of intracellular signalling pathways. The noncanonical activation of Notch signalling represents a crosstalk between Notch signalling and other intracellular signalling pathways (see above). Interactions between Notch and the Wnt pathway have been best characterized, but other interactions with various pathways have also been described.

- Wnt-initiated signalling is mediated through the downstream *β*-catenin [26]. The Wnt and Notch pathways seem to act in synergy to maintain the stem cell pool [26, 27]. The crosstalk between these two pathways seems to occur at both the intracellular level and between cells in the stem cell niche. Firstly, members of the Wnt pathway regulate the expression of established Notch target genes, and inhibition of Wnt signalling affects the expression of both Wnt and Notch target genes [20, 28]. Secondly, Wnt signalling can affect the expression of Notch1 as well as HoxB4 [29]. The HoxB4 transcription factor is important for HSC self-renewal and expansion by inducing the expression of genes preferentially expressed by HSCs and downregulating genes associated with myeloid differentiation [30, 31]. Finally, an example of extrinsic crosstalk between these two pathways in the stem cell niche is the induced expression of Notch ligands by activated *β*-catenin in stromal cells which thereby induce-Notch-mediated intracellular signalling in adjacent HSCs [32].
- Notch signalling becomes a part of a more extensive network through its crosstalk with the Wnt pathway that interacts with several other intracellular pathways [27], including (i) Hedgehog signalling [33], (ii) Prostaglandin E2 signalling; animal experiments suggest that this crosstalk is dependent on a protein kinase A-dependent mechanism that connects the pathways via *β*-catenin [34], (iii) Transforming growth factor-*β* (TGF-*β*) and Bone morphogenic protein (BMP) signaling which targets the common intracellular mediator Smad4 that directly interacts with members of the Hox transcription factor family [26, 35], and (iv) Angiopoietin-1/Tie2 signalling which is also important in HSCs [36]; this signalling targets Cdh2 (N-cadherin) [37] that seems to activate *β*-catenin signalling through protein kinase B (Akt-) dependent mechanisms [38].
- Hey2 is a transcription factor that seems to act downstream of Notch in primitive hematopoietic cells, and studies in zebrafish suggest that its expression is maintained by Hedgehog as well as Vascular endothelial growth factor signalling [39].
- Several members of the NF-*κ*B family (including p65, p50, RelB, and c-Rel) are under transcriptional control by Notch-initiated signaling, and decreased levels were found in Notch-1 antisense transgenic (Notch-AS-Tg) mice [40]. NF-*κ*B is an important regulator of the expression of several chemokines, and Notch-initiated signalling may thereby affect chemotaxis and cell trafficking [41, 42].
- The Ets transcription factor Er71 seems to be a common downstream target both for the Wnt, Notch and BMP signalling pathways [43].

These observations clearly illustrate that Notch signalling is part of an extensive network of interacting pathways. These pathways are important for normal HSCs, and several of them are also important in the development of myeloid malignancies. Besides Notch signalling pathways (see below), the extensive network of interacting pathways includes the Wnt pathway [44, 45], Ang-1/Tie2 [46], HoxB4 [44, 47], Hedgehog signalling [44], BMP [35], NF-*κ*B [48], and TGF-*β*/Smad4 [35, 49] signaling pathways. Thus, Notch signalling is a part of an extensive network involving several interacting pathways both in normal and leukemic hematopoietic cells.

## 4. Notch Signalling in the Immune System

### 4.1. The Role of Notch in T Cell Development

 Notch signalling is directly involved in the regulation of thymic T cell development with Notch1 acting as a key receptor responsible both for the lineage commitment and inhibition of other differentiation directions [1]. The DLL4 ligand is expressed by thymic epithelial cells and is essential for T lineage commitment [50] (Table 1). The *αβ* T cell development depends on Notch signalling, and transition through the *β*-selection checkpoint is then dependent on both Notch signalling [1] as well as CXCL12 ligation of CXCR4 with initiation of PI3K signalling [51]. Notch1 expression is downregulated after *β*-selection [1]. In contrast, the *γδ* T cell development seems less dependent on Notch signalling.

### 4.2. Effects of Notch on Peripheral T Cell Subsets

 Naive T cells exit the thymus and migrate to the periphery where they mediate immune responses after antigenic recognition together with adequate costimulation. Activated naive CD4^+^ T helper cells (Th0 cells) can differentiate towards Th1, Th2, Th9, Th17, and Th22 helper cells, or they may alternatively develop into induced regulatory T (iTreg) cells that act together with thymus-derived natural T regulatory (nTreg) cells to inhibit immune responses. Activation of naive CD8^+^ T cells leads to the differentiation towards cytotoxic T lymphocytes (Tc, or also called CTLs). A detailed list of possible interactions between ligand-presenting and receptor-expressing cells involved in normal hematopoiesis, T cell development, and T cell activation is given in Table 1.

#### 4.2.1. Tc Cells

The transcriptional regulator eomesodermin (Eomes) regulates the expression of perforin and granzyme B in CD8^+^ cytotoxic T cells [56]. Notch1 seems to directly regulate expression of Eomes as well as perforin and Granzyme B by binding to their promoters, and *γ*-secretase inhibitors (GSIs) thereby attenuate in vitro T cell cytotoxicity. In addition, Notch2-ICD seems to cooperate with CREB1 in the regulation of granzyme B expression [57].

#### 4.2.2. Th1 and Th2 Cells

Th1 cells produce IFN*γ* while Th2 cells produce IL-4, IL-5, and IL-13 as their signature cytokines. Jagged ligands expressed by APCs are important for Th2 differentiation whereas DLL ligands (DLL1 and/or DLL4) seem to promote Th1 and inhibit Th2 differentiation, but the additional molecular events in this differentiation have not been characterized [1]. Thus, it is not known which Notch receptors mediate the DLL-induced Th1 cell differentiation signal. Although canonical Notch signalling does not seem to be essential [1], Notch3 signalling is possibly also involved in Th1 differentiation [58]. On the other hand, Th2 cell differentiation seems to involve CBF1 and IL-4, as well as the Th2-specific transcription factor Gata3 that is a Notch target gene [58].

#### 4.2.3. Th17 Cells

These cells represent a proinflammatory subset distinct from Th1 cells; their signature cytokines are IL-17A, IL-21, and IL-22, and they express the Th17-specific transcription factor ROR*γ*. DLL ligands might play a role in the generation of Th17 cells [1], and recent in vitro studies [54] suggest that DLL4 inhibits Th2 cytokine production, contributes to Th17 differentiation, and upregulates ROR*c* expression. Both the ROR*c* and IL-17 gene promoters then seem to be direct targets for Notch-initiated signalling.

#### 4.2.4. Treg Cells

Both natural (nTreg) and peripherally induced Treg (iTreg) cells are important for downregulation of immune responses. FoxP3 is a Treg-specific transcription factor, and the cells typically release IL-10. Notch ligands (usually the Jagged family) increase Treg cell differentiation in vitro [1, 8, 59, 60], but this differentiation is not Notch-dependent because Notch loss-of-function mutant mice do not lack Treg cells [1]. Notch1 signalling seems to contribute to the FoxP3 expression [61], and Notch3 receptors are increased on murine CD4^+^CD25^+^ Treg cells [62]; exposure to JAG2-overexpressing hematopoietic progenitors seems to increase the expression of Notch3 and FoxP3 in Treg cells [63].

### 4.3. Notch and Autoimmunity

 There are few reports of Notch in human autoimmune diseases. Recently Jiao et al. [64] reported increased expression of Notch2/3/4 in Th cells from patients with active rheumatoid arthritis, and the increased expression of Notch3 was mainly detected in activated T cells. These patients also show increased nuclear translocation of NICD in Th cells as well as increased expression of the Notch target gene HES-1 [64]. Another recent study [65] reported increased expression of Notch1/3 and decreased Notch2 together with downregulated DLL1 among peripheral blood mononuclear cells from patients with autoimmune thrombocytopenia. Finally, Sodsai et al. [66] suggest that defective Notch1 upregulation during T cell activation is important for increased disease activity in patients with systemic lupus erythematosus. However, it should be emphasized that a major part of these studies only included a description of Notch/Notch ligand expression in immunocompetent cells; it is therefore difficult to judge whether this altered expression is directly involved in disease development/progression or only represents secondary (innocent bystander or secondary) effects that may not be clinically important. 

 The importance of Notch signalling in autoimmune diseases has been investigated more in detail in murine models of autoimmune disorders, and these results are summarized in Table 2. The contribution of Notch signalling has especially been investigated in experimental autoimmune encephalomyelitis (EAE), an experimental model of multiple sclerosis. Th1 and Th17 cells are important for the development of this disease, and inhibitions of Notch3, *γ*-secretase, or DLL1 inhibit proinflammatory Th1/Th17 responses and improve disease symptoms. In contrast, DLL2-initiated signalling increases symptoms whereas JAG1 results in improvement. Furthermore, Notch1-induced signalling also seems important for the development of autoimmunity in other organs, but these effects seem to differ depending on the experimental model. Finally, Notch-induced signalling with induction of Treg cells can inhibit the development of autoimmune diabetes in various disease models. It should be emphasized that several of these conclusions are based on the observed effects when using specific Notch inhibition in the experimental models (like specific neutralizing antibodies) as described in detail in Table 2; these effects of specific inhibitors demonstrate that Notch-initiated signalling is directly involved in the regulation of experimental murine autoimmunity. 

 Many clinical and laboratory features of GVHD, especially in its chronic form, resemble those of autoimmune diseases, and the pathophysiological mechanisms also seem to show similarities [72, 73]. Autoimmune phenomena can be seen after both auto- and allotransplantation, and the most common manifestations seem to be thyroid disease and autoimmune cytopenias. Certain manifestations of GVHD have been postulated to represent a specific loss of tolerance to self structures. Taken together, these observations suggest that the role of Notch signalling in autoimmune diseases is relevant also for development of GVHD.

## 5. Immune Reconstitution after Stem Cell Transplantation

Immunological reconstitution after stem cell transplantation has been extensively reviewed previously [74–78], and the most important observations both in allogeneic and autologous transplantation are (i) early lymphoid reconstitution is associated with a decreased risk of relapse, suggesting that antileukemic immune reactivity is mediated early after transplantation [79–82] and (ii) a long-lasting quantitative CD4 T cell defect can persist for several months post transplant [75, 76, 78]. 

 The quantitative CD4^+^ T cell defect after allotransplantation may last for several months, but early normalization seems more common after reduced intensity conditioning [78]. Infusion of a high number of CD4^+^ T cells and Natural Killer (NK) T cells seems to be associated with a better prognosis [83], an observation supporting the hypothesis that early antileukemic immune reactivity is important. Recovery of dendritic cells occurs more slowly [84], and an abnormal ratio between various dendritic cell subsets may persist for months after transplantation [84]. Low dendritic cell counts one month post transplant also seem to be an independent adverse prognostic factor for the overall survival [85]. 

 In autotransplanted patients the immunological reconstitution differs between patients receiving peripheral blood and bone marrow autografts [86]. Mobilized stem cells are now most commonly used, and during the first posttransplant months the patients generally show early recovery of CD8^+^ T cells, CD14^+^ monocytes, and CD56^+^ NK cells [86–88]. Circulating dendritic cells are usually normalized relatively early, although differences in dendritic cell subset composition may persist for several months also in these patients [88]. The T cell defect is detected after 6 months for most of these patients and may last for more than a year [87], and it is mainly due to reduced naive CD3^+^CD4^+^CD45RA^+^ T cells, but reduced CD8^+^ naive T cells can also be seen [87, 88].

## 6. Notch in Hematological Malignancies

### 6.1. T-Lineage Acute Lymphoblastic Leukemia

 ALL is characterized by accumulation of immature lymphoblast of either B or T cell lineage origin in bone marrow and eventually other lymphoid organs. T-ALL accounts for approximately one third of all cases [89]. Notch involvement in T-ALL was first described in patients with the rare t(7;9) (q43;q34.3) translocation that leads to the expression of a cytoplasmic form of the Notch1 receptor with constitutive activity [90]. However, the most common Notch abnormality in T-ALL is mutations in the Notch1 alleles that result in constitutive activation of the pathway; this is seen in more than half of the patients [91]. These Notch1 mutations are located at specific hotspots and affect critical negative regulatory elements of the protein. The molecular mechanisms by which aberrant Notch1 signalling contributes to T-cell transformation are not yet fully understood. Oncogenic Notch1 probably cooperates with oncogenic transcription factors such as c-Myc [92], E2A-PBX [93], and Ikaros [94], but the aberrant Notch1 signalling is not sufficient for leukemic transformation [95]. Observations in animal models suggest that even nonmutational Notch1 activation contributes to leukemogenesis [96], probably through activation of c-Myc that is a direct downstream target of Notch1 [92]. Finally, the prognostic impact of Notch1 mutations was demonstrated in recent clinical studies where the mutations were associated with good prognosis both in children [97] and adults [98].

### 6.2. Acute Myeloid Leukemia

 The prevalence of Notch mutation in AML is probably less than 5% [99, 100], and Notch ligation in AML cells has diverse or only minor effects [101]. AML cells seem to express JAG1, Notch1 and Notch2 [102–104]. In hematopoiesis, Notch1, drives myeloid differentiation through the expression of transcriptional factor PU.1. Results obtained from Chen et al. in [104] show that the Notch1 gene and protein expression were decreased in human AML samples in comparison with normal hematopoietic stem cells. This decrease of Notch1 expression was associated with a concordant downregulation in PU.1, suggestive of impeded PU.1-mediated myeloid signalling and thus contributing to AML leukemogenesis [104]. However, gene expression profiling of primary human AML cells has identified a subgroup of patients with recurring mutations in Notch [105, 106]; the expression profile of these patients seems to be mainly determined by silencing of the CEBPA gene through promoter hypermethylation [106]. The CEBPA gene encodes for the transcription factor CCAAT/enhancer-binding protein alpha (C/EBPalfa); this gene is mutated in approximately 10% of AML cases [107]. AML cells with silenced CEBPA gene and Notch mutations cluster together [106].

### 6.3. Chronic Lymphocytic Leukemia

 Chronic lymphocytic leukemia (CLL) is characterized by detection of malignant CD5^+^CD19^+^ B cells in blood, bone marrow, and eventually other lymphoid organs. Several recent studies suggest that the Notch system is important also in B-CLL. Firstly, B-CLL cells express high levels of Notch2 that regulate the expression of antiapoptotic CD23a [108]. Secondly, Notch1/2 and the ligands JAG1/2 are also constitutively expressed in B-CLL [109] and are then associated with resistance to apoptosis [109]. Upregulation of Notch1 is observed during treatment with the MDM2/p53 inhibitor Nutlin-3 and possibly represents a feedback mechanism involved in restrain of the Nutlin-3 effects [110].

## 7. Possible Strategies for Notch Targeting in Patients Treated with Stem Cell Transplantation

### 7.1. Therapeutic Tools for Inhibition of Canonical Notch Signalling

 An overview of possible therapeutic tools is given in Table 3. The tools include ligands, agonistic and antagonistic antibodies, stimulatory fusion proteins, and inhibitors of intracellular signalling. Inhibition of the *γ*-secretase activity with general downregulation of Notch signalling has been used in experimental in vitro studies [111]. Unless NICD is translocated to the nucleus, the NICD form of Notch is ubiquitinated and thereafter degraded by the proteasomes; and proteasomal inhibitors may thus enhance Notch signalling. Various proteasomal inhibitors are now used in the treatment of hematologic malignancies, and they are also tried as immunosuppressive agents [112–115], but it is not known whether inhibition of Notch signalling contributes to their clinical effects.

 The results summarized in Table 3 suggest that if a detailed characterization of the immune system is available, it would be possible to design therapeutic strategies based on stimulation or inhibition of selected Notch-mediated effects. Especially in allograft recipients, Notch inhibition may offer the opportunity to combine immunosuppressive GVHD prophylaxis with direct antileukemic effects.

### 7.2. Notch-Driven Stem Cell Expansion Effects on Posttransplant Myeloid Reconstitution

 The delayed hematopoietic stem cell engraftment commonly seen after cord blood transplantation is probably due to an inadequate numbers of progenitor cells in the graft [116]. In a recent phase I clinical trial, CD34^+^ cord blood cells were cultured *ex vivo* with the extracellular DLL1 domain fused to the Fc domain of human IgG. After 16 days and following myeloablative conditioning, the expanded cells were transplanted together with an unmanipulated allograft. No unexpected toxicity was observed, and the patients showed early myeloid reconstitution with a shortened time until peripheral blood neutrophil counts ≥0.5 × 10^9^/L (16 days compared with 26 days for the controls). In four of these patients, the neutrophil reconstitution was attained at a time when at least 80% of the cells were derived from the manipulated grafts, and for two patients, long-term persistence of these cells was documented after 180 and 240 days. For other patients, persisting cells were derived from the unmanipulated graft. Thus, targeting of Notch can be used for ex vivo stem cell expansion of allogeneic stem cells. Whether a similar methodological approach can be used for ex vivo expansion of autologous stem cells in poor mobilizers has not been investigated, but the use of a CXCR4 antagonist would at present be the first alternative for such patients [125]. Another possible therapeutic strategy may be in vivo expansion of stem and progenitor cells by administration of the Delta1-IgG preparation.

### 7.3. Notch Targeting and Posttransplant Lymphoid Reconstitution

 Early lymphoid reconstitution after stem cell transplantation is associated with decreased relapse risk in several hematological malignancies [126]. The study by Delaney et al. [116] demonstrated that ex vivo stem cell expansion reduced the time until neutrophil reconstitution, but the manipulated grafts did not contain T cells, and long-term T cell engraftment was always derived from the unmanipulated grafts (see above). Notch-induced signalling is also important for T lymphopoiesis, but it is not known whether alternative ex vivo or in vivo strategies for Notch targeting can be used to increase lymphopoiesis and thereby shorten the time until lymphoid reconstitution and thereby shorten the posttransplant CD4 defect. Such a strategy may become useful in autotransplanted patients to increase posttransplant antileukemic T cell reactivity, whereas it would be more difficult to use in allotransplanted patients with the risk of severe and potentially lethal GVHD.

### 7.4. Notch Targeting of Malignant Hematopoietic Cells—The Initial Clinical Experience

 Allogeneic and autologous stem cell transplantation is mainly used in the treatment of hematologic malignancies, and Notch signalling seems important in these diseases, especially T-ALL (see above). Inhibitors of the *γ*-secretase activity have been developed, but the initial clinical Phase I studies in T-ALL patients showed a low efficiency and severe gastrointestinal toxicity [127]. Several other Notch-targeting drugs are now being developed for use in clinical phase 1-2 trials, and one of them has also been investigated in a phase 3 trial [128]. These agents are mainly *γ*-secretase inhibitors that are tried in the treatment of various cancers. Whether these inhibitors will have an acceptable toxicity and higher efficiency has to be addressed in future studies.

### 7.5. Targeting Noncanonical Notch Signalling—Possible Mechanisms for the Antileukemic Effects of Several Targeted Therapies

 Several drugs may affect the expression of Notch-targeted genes through inhibition of the noncanonical pathways such as HSP90, HDAC, PI3K/Akt/mTOR, and proteasomal inhibitors [2, 10]. Some of these drugs may have combined effects; for example, proteasomal inhibitors may alter noncanonical signalling through NF*κ*B inhibition together with decreased degradation of the NICD form involved in canonical signalling. Furthermore, specific PI3K inhibitors are now evaluated in clinical studies [129]. The PI3K-Akt pathway is upstream to mTOR, and the mTOR inhibitor rapamycin is used for immunosuppression after allotransplantation and is also being investigated as an anticancer agent in hematologic malignancies. Thus, Notch inhibition may contribute to the efficiency of several new anticancer and/or immunosuppressive-targeted therapeutics.

### 7.6. Notch Targeting in Stem Cell Recipients: Immunosuppression versus Immunostimulation

 As can be seen from Table 2, Notch targeting can be used both for immunostimulation and immunosuppression in experimental autoimmunity, and these observations may be relevant also for human GVHD [72, 73]. Thus, Notch signalling seems important both for T lymphopoiesis and for regulation of the peripheral T cell system. Notch agonists may thus become useful to enhance T cell reconstitution after both allogeneic and autologous stem cell transplantation. Early lymphoid reconstitution is then associated with decreased risk of cancer relapse, and earlier T cell reconstitution would possibly further reduce the relapse risk. T cell defects are in addition associated with an increase of severe opportunistic infections especially in allotransplant recipients [130, 131], and early reconstitution may also reduce this risk. On the other hand, enhancement of T cell reconstitution after allotransplantation has to be balanced against a possible risk of inducing severe and potentially lethal GVHD. 

 A second possibility could be to use Notch-targeting therapy to modulate the function of peripheral T cells. Immunostimulatory agonists could then be used to enhance antileukemic immune reactivity after autologous stem cell transplantation. Clinical studies have demonstrated that antileukemic T cells can be detected in autotransplanted leukemia patients and that this reactivity can possibly be enhanced by vaccination therapy [126]. Immunostimulatory Notch targeting may then increase this antileukemic reactivity and possibly increase the efficiency of anticancer vaccines. 

 A third strategy is to consider immunomodulatory strategies to reduce the risk of severe GVHD after allotransplantation. Yvon et al. [132] overexpressed the JAG1 ligand in alloantigen-presenting B cells and observed induction of Treg cells from CD45-RA^+^ T cells; these allospecific Treg cells caused a specific inhibition of proliferative and cytotoxic T cell responses against the priming alloantigens. Thus, Notch agonists may be used to induce specific tolerance against alloantigens, and both ex vivo generation of immunoregulatory cells and in vivo administration of agonists should be considered. An alternative would be to use Notch inhibition for suppression of effector T cells (see Table 2). However, the use of Notch inhibition in targeting the peripheral T cell system seems less attractive because this approach may interfere with lymphoid reconstitution and aggravates the posttransplant T cell defects (see above).

### 7.7. Mesenchymal Stromal Cells

 Multipotent mesenchymal stromal cells (MSCs), also called mesenchymal stem cells, are able to differentiate into a variety of cell types including osteoblasts, chondrocytes, and adipocytes [133]. These cells are important components of the bone marrow HSC niche and can support HSC maintenance and engraftment [134]. Intriguingly, MSCs have also been shown to have immunomodulatory properties which are of value in a clinical setting with regard to treatment of GVHD after hematopoietic stem cell transplantation (HSCT). Moreover, cotransplantation of HSCs and MSCs can facilitate hematopoietic engraftment and was shown to accelerate lymphocyte recovery in clinical HSCTs [135]. The exact mechanisms of how MSCs contribute to hematopoietic reconstitution remain unclear though both immunomodulatory effects as well as effects on HSC self-renewal capacity are assumed and Notch signalling has been implicated in these effects. Studies of Notch function have revealed that Notch signalling affects various differentiation capabilities of MSC, including differentiation in direction of osteoblasts [17, 136, 137]. Notch signalling in bone marrow is suggested to maintain a pool of mesenchymal progenitors by suppressing osteoblast differentiation [16]. In addition, Notch signalling has been identified as a possible pathway involved in osteogenic differentiation of MSCs induced by soluble mediators derived from endothelial cells [138]. 

 The infusion of MSCs has been tried in the treatment of GVHD. There seems to be a consensus that these cells are immunomodulatory, but the initial clinical studies have shown conflicting results with regard to the efficiency of MSC in the treatment of GVHD [139–141]. Human bone marrow-derived MSCs were found to express high levels of functionally active toll-like receptors (TLR) 3 and 4, and these cells had an immunosuppressive effect on T-cell proliferation after ligation of either TLR3 or TLR4. Suppression of T-cell activation was inhibited by neutralization of JAG1 and inhibition of *γ*-secretase activity, thus implying a role of impaired Notch receptor signalling in T cells [142]. Additional mechanisms possibly involved in MSC-induced immunomodulation could be interactions with the NK cell system, inhibition of dendritic cell differentiation, or modulation of the humoral system [139]. 

 To conclude, even though additional studies are definitely needed, these studies suggest that Notch signalling may be important both for the development of supportive cells in stem cell niches and for the immunomodulatory/GVHD-suppressing effect of the MSC.

### 7.8. Concluding Remarks

 The Notch ligand/receptor system is important for (i) development and regulation of the T cell system and (ii) regulation of normal as well as leukemic hematopoiesis. The final biological effects of Notch targeting in stem cell recipients are difficult to predict, and depend both on the involved ligand(s) and receptors, and signalling through the canonical intracellular pathway is modulated by noncanonical signalling. The interactions between Notch-initiated signalling and several other intracellular signalling pathways further make it difficult to predict the final effect of Notch-targeted therapy. Pharmacological tools for targeting of Notch-mediated signalling are now being developed. However, because the effects of Notch-targeted therapy are difficult to predict a more detailed study of the post-transplant hematopoiesis as well as the T cell system is necessary before clinical studies of these agents in transplant recipients can be designed. However, such studies should be encouraged because Notch targeting may represent a unique possibility to combine enhancement of reconstitution, immunomodulation, and direct anticancer treatment.

## Figures and Tables

**Table 1:** Notch ligand-receptor interaction: a summary of possible interactions involved in normal hematopoiesis, T cell development, and regulation of the peripheral T cell system.

SIGNAL-INITIATING CELL		SIGNAL-RECEIVING CELL		
Cell type	Ligand	Cell type	Receptor	References
*Bone marrow stromal cells*		*Bone marrow stem cells*		
Osteoblasts (endosteal niche)	JAG1	Sca-1^+^, c-kit^+^, Lin^−^	Notch1	[13]
	JAG1, JAG2	Sca-1^+^, c-kit^+^, CD117^+^	Notch1, Notch2	[52]
Endothelial cells (vascular niche)	JAG1, JAG2, DLL4, DLL1	Sca-1^+^, c-kit^+^, Lin^−^	Notch1, Notch2	[24]

*Thymic epithelial cells (TECs)*		*T cell progenitors*		
TECs	DLL4	Thymocytes	Notch1	[1, 50]

*Antigen-presenting cells*		*Peripheral T cells*		
APC	DLL1	Tc	Notch1, Notch2	[1]
DC	DLL1, DLL4	Th1	Notch3	[1, 8, 53]
DC	JAG1, JAG2	Th2	Notch1, Notch2	[1, 8, 53]
APC	DLL1, DLL4	Th17	Notch	[1, 54]
pDC	DLL4	Th1 IL-10^+^	Notch	[55]

Abbreviations: Delta-like (DLL); Jagged (JAG); Thymic epithelial cells (TECs); Antigen presenting cells (APC); Dendritic cells (DC); plasmacytoid DC (pDC); Cytotoxic T cells (Tc); Helper T cells (Th); Interleukin-10 (IL-10).

**Table 2:** Therapeutic targeting of Notch in murine autoimmunity *in vivo*.

Disease and intervention	Therapeutic effect	References
*Experimental autoimmune encephalomyelitis (EAE; model of multiple sclerosis)*		
*γ*-secretase inhibitor	Inhibition of disease-associated Th1 responses and improvement of symptoms	[8, 58, 67]
Notch1 neutralizing antibodies	No effect on Th1 and Th17 responses	[8, 58]
Notch3 neutralizing antibodies	Decreased Th1 and Th17 responses, inhibition of the ability of myelin-primed T cells to transfer the disease	[8, 58]
DLL1 neutralizing antibodies	Reduced Th1 responses and EAE symptoms	[8, 68]
Activating DLL1-Fc fusion protein	Increased Th1 responses and EAE symptoms	[8, 68]
Neutralizing JAG1 antibodies	EAE disease progression	[8, 68]
Activating JAG1-Fc fusion protein	Improvement of EAE symptoms	[8, 68]

*Experimental hepatitis*		
*γ*-secretase inhibitor	Reduced Notch1 signalling and FoxP3 expression, spontaneous hepatic lymphocyte infiltration consistent with autoimmune hepatitis (C57BL/6 mice)	[61]

*Murine diabetes*		
Lck-Notch3-IC transgenic mice	Up regulation of the generation and function of CD4^+^CD25^+^ Treg. The mice failed to develop streptozotocin-induced autoimmune diabetes. Adoptive transfer of the *lck*-Notch3-IC transgenic CD4^+^ cells to wild-type recipients prevented the progression of the disease.	[62]
Exposure to JAG2-expressing hematopoietic progenitor cells	Activation of Notch3 signalling with increased Treg proliferation and prevention of diabetes in NOD mice.	[63]

*Multiorgan autoimmune disease*		
Loss of functional mutation in the Itch ubiquitin ligase	This ligase is involved in Notch1 degradation; homozygous mice develop an autoimmune-like disease mainly affecting lungs, skin, and lymphoid organs.	[2, 69–71]

Abbreviations: Experimental autoimmune encephalomyelitis (EAE), Delta-like (DLL), Jagged (JAG), Helper T cells (Th).

**Table 3:** Potential molecular tools for targeting of Notch signalling.

Molecular tool	Observations in clinical or experimental studies	References
*Natural receptor ligands*		
JAG2-expressing hematopoietic progenitor cells	Activation of Notch3 signalling with increased Treg proliferation and prevention of diabetes in NOD mice.	[63]
Fusion proteins of natural ligands and Fc-Ig	DLL1-IgG-Fc has been used for expansion of human umbilical cord stem cell expansion; cells caused no unexpected toxicity and contributed to long-term engraftment. Could be used for transplantation.	[116]
JAG1- and DLL2-Fc fusion proteins	Both types of fusion proteins have shown immunomodulatory effects in experimental murine disease models.	[8, 68]

*Receptor- or ligand-directed antibodies*		
Agonistic antibodies	Agonistic antibodies have been identified for their reactivity against Notch2 or Notch3.	[117, 118]
Antagonistic antibodies directed against Notch	Several antagonistic Notch1-, Notch2- or Notch3-directed antibodies have been tested in vitro and in vivo.	[8, 58, 117, 119, 120]
Antagonistic antibodies directed against Notch ligands	Both DLL- and JAG1- specific antibodies show immunomodulatory effects in murine disease models.	[8, 68]
DLL1 neutralizing antibodies	Reduced Th1 responses and improvement of EAE symptoms.	[8, 68]
DLL4 neutralizing antibodies	In CSC-driven colon and breast xenograft models, anti-human DLL4-blocking antibodies inhibited tumour growth and reduced tumour-initiating cell frequencies.	[121]
Antibodies that inhibit the transcription-regulating complex	Development of such antibodies could inhibit parts of the Notch-initiating effects and possibly limit the toxicity.	[122]
Activating JAG1-Fc fusion protein	Improvement of EAE symptoms.	[8, 68]
Activating DLL2-Fc fusion protein	Increased Th1 responses and progression of EAE symptoms.	[8, 68]

*Inhibition of intracellular signalling*		
*γ*-secretase inhibitor	Reduced Notch1 signalling and FoxP3 expression in Treg cells in murine disease models.	[58, 61, 67]
Proteasome inhibitors	Inhibition of noncanonical Notch signalling.	[2, 10, 123, 124]
Inhibition of the PI3K-Akt-mTOR pathway	Inhibition of noncanonical Notch signalling.	[2, 10, 48]
Peptide that inhibits assembly of the transcription-regulating complex	The small hydrocarbond-staple peptide SAHM1 inhibits Notch signalling *in vitro*.	[122]

Abbreviations: Experimental autoimmune encephalomyelitis (EAE), Delta-like (DLL), Jagged (JAG), Helper T cells (Th), Cancer stem cell (CSC).
